# Supplementary material for: Ectopic Expression of Sugarcane ScAMT1.1 Has the Potential to Improve Ammonium Assimilation and Grain Yield in Transgenic Rice under Low Nitrogen Stress
Source: Int J Mol Sci. 2023 Jan 13;24(2):1595. doi: 10.3390/ijms24021595 (PMC9863325; doi:10.3390/ijms24021595)
Supplement: Supplementary file 1 [file ijms-24-01595-s001.zip › Supplementary Figure S1.pdf]

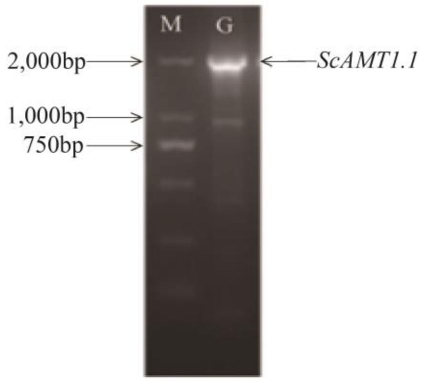

Supplementary Figure S1 Electorphoresis map of PCR products of sugarcane *ScAMT1.1*.
Note: M, 2,000bp DNA marker. G, sugarcane *ScAMT1.1*.
