# Supplementary material for: Ectopic Expression of Sugarcane ScAMT1.1 Has the Potential to Improve Ammonium Assimilation and Grain Yield in Transgenic Rice under Low Nitrogen Stress
Source: Int J Mol Sci. 2023 Jan 13;24(2):1595. doi: 10.3390/ijms24021595 (PMC9863325; doi:10.3390/ijms24021595)
Supplement: Supplementary file 1 [file ijms-24-01595-s001.zip › Supplementary Figure S2.pdf]

```
1     TTCGAGCTCGGTACCCGGGGATCCGATTCACCCTCCCAATCCCATCGCGTCTCCATACCTCTCTTATTACGCCAC
76    GCCGGAGGAGGGACAGAGAACGAGCGAACCCAAGCTAAGATGTCGACGTGCGCGGCGGACCTGGCGCCGCTGCTG
                                              M  S  T  C  A  A  D  L  A  P  L  L 
151   GGCCCGGCGGCGGCGAACGCCACGGACTACCTCTGCGGCCAGTTCGCGGACACGACGTCCGCGGTGGACGCCACG
       G  P  A  A  A  N  A  T  D  Y  L  C  G  Q  F  A  D  T  T  S  A  V  D  A  T 
226   TACCTGCTCTTCTCGGCCTACCTCGTCTTCGCCATGCAACTCGGCTTCGCCATGCTCTGCGCCGGCTCCGTCCGC
       Y  L  L  F  S  A  Y  L  V  F  A  M  Q  L  G  F  A  M  L  C  A  G  S  V  R 
301   GCCAAGAACACCATGAACATCATGCTCACCAACGTGCTCGACGCCGCCGCGGGAGCGCTCTTCTACTACCTCTTC
       A  K  N  T  M  N  I  M  L  T  N  V  L  D  A  A  A  G  A  L  F  Y  Y  L  F 
376   GGCTTCGCCTTCGCCTTCGGCACGCCCTCCAACGGCTTCATCGGGAAGCAGTTCTTCGGGCTCCAGCAGCTGCCC
       G  F  A  F  A  F  G  T  P  S  N  G  F  I  G  K  Q  F  F  G  L  Q  Q  L  P 
451   AAGACCGGCTTCGATTACGACTTCTTCCTCTACCAGTGGGCCTTCGCCATCGCCGCCGCGGGCATCACGTCGGGC
       K  T  G  F  D  Y  D  F  F  L  Y  Q  W  A  F  A  I  A  A  A  G  I  T  S  G 
526   TCCATCGCCGAGAGGACCCAGTTCGTTGCCTACCTCATCTACTCCGCGTTCCTCACGGGGTTCGTGTACCCCGTG
       S  I  A  E  R  T  Q  F  V  A  Y  L  I  Y  S  A  F  L  T  G  F  V  Y  P  V 
601   GTGTCCCACTGGTTCTGGTCCGCCGACGGCTGGGCCGCCGCCAGCCGAACGTCCGGCCCGCTGCTCTTCGGGTCC
       V  S  H  W  F  W  S  A  D  G  W  A  A  A  S  R  T  S  G  P  L  L  F  G  S 
676   GGCGTCATCGACTTCGCGGGCTCCGGTGTCGTCCACATGGTCGGCGGCATCGCGGGGCTCTGGGGCGCGCTTATC
       G  V  I  D  F  A  G  S  G  V  V  H  M  V  G  G  I  A  G  L  W  G  A  L  I 
751   GAGGGCCCCCGCATCGGGCGCTTCGACCACGCCGGCCGCTCCGTGGCGCTCAAGGGCCACAGCGCGTCGCTCGTG
       E  G  P  R  I  G  R  F  D  H  A  G  R  S  V  A  L  K  G  H  S  A  S  L  V 
826   GTGCTTGGCACCTTCCTGCTGTGGTTCGGCTGGTACGGGTTCAACCCCGGGTCCTTCACCACCATCCTCAAGTCC
       V  L  G  T  F  L  L  W  F  G  W  Y  G  F  N  P  G  S  F  T  T  I  L  K  S 
901   TACGGCCCCGCGGGCACCGTCCACGGGCAGTGGTCGGCCGTGGGCCGCACCGCCGTCACCACCACCCTCGCCGGC
       Y  G  P  A  G  T  V  H  G  Q  W  S  A  V  G  R  T  A  V  T  T  T  L  A  G 
976   AGCGTCGCCGCGCTCACCACGCTGTTCGGGAAGCGGCTCCAGACGTGCCACTGGAACGTGGTGGACGTCTGCAAC
       S  V  A  A  L  T  T  L  F  G  K  R  L  Q  T  C  H  W  N  V  V  D  V  C  N 
1051  GGCCTCCTCGGCGGGTTCGCGGCCATCACGGCCGGGTGCAGCGTGGTGGAGCCGTGGGCGGCCGTCATCTGCGGC
       G  L  L  G  G  F  A  A  I  T  A  G  C  S  V  V  E  P  W  A  A  V  I  C  G 
1126  TTCGTGTCCGCGTGGGTGCTCATCGGCGCCAACGCGCTCGCCGCGCGCCTCAAGTTCGACGACCCGCTGGAGGCG
       F  V  S  A  W  V  L  I  G  A  N  A  L  A  A  R  L  K  F  D  D  P  L  E  A 
1201  GCGCAGCTGCACGGCGGGTGCGGCGCCTGGGGAGTCCTCTTCACGGGCCTCTTCGCGAGGCAAAAGTACGTGGAG
       A  Q  L  H  G  G  C  G  A  W  G  V  L  F  T  G  L  F  A  R  Q  K  Y  V  E 
1276  GAGATCTACGGCGCCGGGAGGCCCTACGGGCTGTTCATGGGCGGCGGCGGGAAGCTCCTCGCAGCGCAGATCATC
       E  I  Y  G  A  G  R  P  Y  G  L  F  M  G  G  G  G  K  L  L  A  A  Q  I  I 
1351  CAGATCCTGGTGATCGCCGGGTGGGTGAGCTGCACCATGGGCCCGCTCTTCTACGCGCTCAAGAAGCTGGACCTG
       Q  I  L  V  I  A  G  W  V  S  C  T  M  G  P  L  F  Y  A  L  K  K  L  D  L 
1426  CTGCGCATCTCGGCCGACGACGAGATGTCCGGCATGGACCTCACCCGGCACGGCGGCTTCGCGTACGTCTACCAC
       L  R  I  S  A  D  D  E  M  S  G  M  D  L  T  R  H  G  G  F  A  Y  V  Y  H 
1501  GACGAGGACCCCGGTGACAAGGCCGGGGTTGGTGGGTTCATGCTCAAGTCCGCGCAGCACCGTGTCGAGCCGGCG
       D  E  D  P  G  D  K  A  G  V  G  G  F  M  L  K  S  A  Q  H  R  V  E  P  A 
1576  GCGGCGGCGGCGACCAGCAACCAGGTGTAAAAATCAGAAGCAAATTAAAACCGAGAGCTGAAGTTACGTGCTTGC
       A  A  A  A  T  S  N  Q  V  *  K  S  E  A  N  *  N  R  E  L  K  L  R  A  C 
1651  CTTTTTCAGTGTGTCCATGTTTGGTCTCGATCGTATCTGCCGGTACTGTTTGGGCAATACTACTTCGTTCGCCAC
1726  TTCGGAGTCGCAAGAATGAATTTGTGTAAACTATAGGAGAGGTATGGCGACGAAGCACGAATCCATATGACTAGT
1801  AGATCCTCTAGAGTCGACCTGCAGGCATGCAAGCTTTCCCCTATAGTGTCACCTAAATAGCTGGCGTA
```

Supplementary Figure S2 The cDNA sequence and amino acid sequence of *ScAMT 1.1* gene

Note: underlined: ammonium transporter signature
