# Supplementary material for: Ectopic Expression of Sugarcane ScAMT1.1 Has the Potential to Improve Ammonium Assimilation and Grain Yield in Transgenic Rice under Low Nitrogen Stress
Source: Int J Mol Sci. 2023 Jan 13;24(2):1595. doi: 10.3390/ijms24021595 (PMC9863325; doi:10.3390/ijms24021595)
Supplement: Supplementary file 1 [file ijms-24-01595-s001.zip › Supplementary Figure S3.pdf]

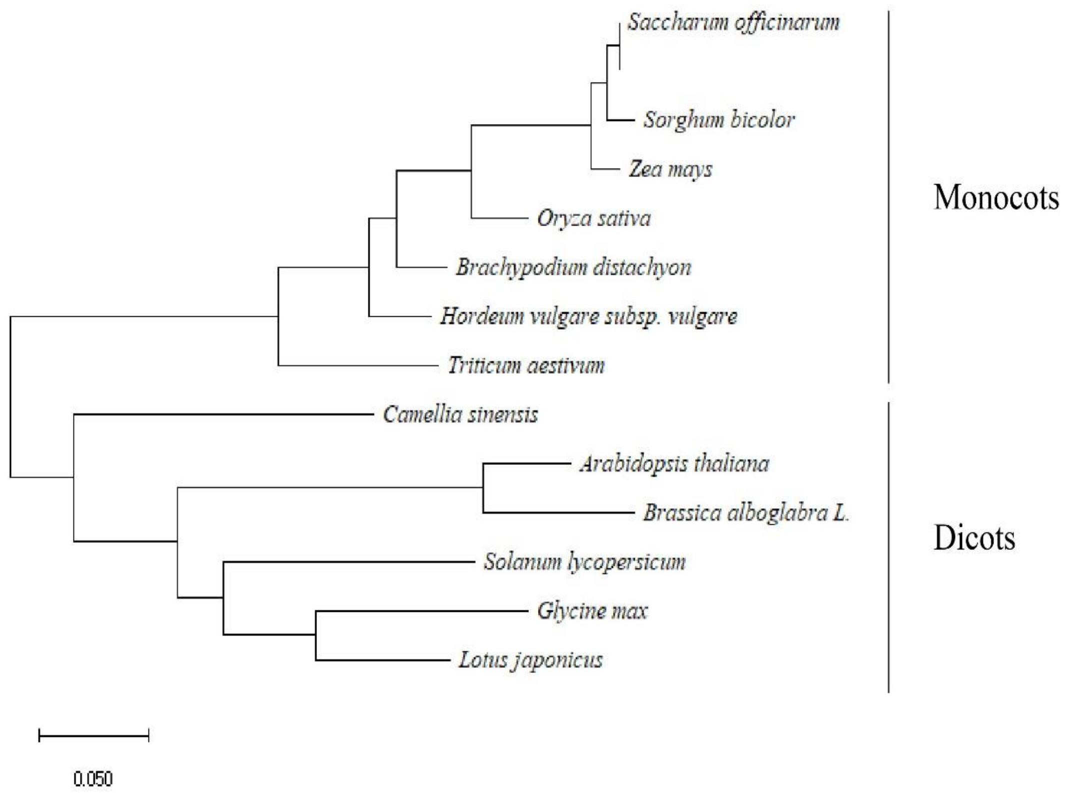

Supplementary Figure S3 Homology analysis of amino acids sequences of ScAMT1.1 and AMT1.1 in other plant species
